# A qualitative study exploring discharge readiness experiences among patients with esophageal cancer undergoing esophagectomy

**DOI:** 10.1080/17482631.2026.2686490

**Published:** 2026-06-09

**Authors:** Yuxin Wang, Ying Wang, Yajing Xue

**Affiliations:** a Department of Minimally Invasive Esophageal Surgery, Tianjin Medical University Cancer Institute and Hospital, National Clinical Research Center for Cancer, Tianjin's Clinical Research Center for Cancer, Tianjin, People's Republic of China

**Keywords:** Esophageal cancer, esophagectomy, discharge readiness, qualitative study, experience

## Abstract

**Purpose:**

To explore the needs and experiences related to discharge readiness among patients after esophagectomy.

**Methods:**

This descriptive qualitative study involved 12 patients who underwent esophagectomy at a tertiary-grade hospital in Tianjin between July and September 2025. Semi-structured interviews were conducted one day prior to discharge. Colaizzi’s seven-step phenomenological analysis method was used to analyze the data.

**Results:**

Three main themes emerged from the analysis: the coexistence of positive and negative emotions related to discharge readiness, perceived dilemmas regarding discharge readiness, and multidimensional challenges in post-discharge home care.

**Conclusions:**

These findings enhance healthcare professionals’ understanding of the feelings and experiences of patients undergoing esophagectomy at the time of discharge. Serious attention should be paid to patients’ emotions, self-care skills, and confidence in home rehabilitation. Personalized intervention strategies are crucial for effectively enhancing discharge readiness. This study provides a basis for conducting effective interventions to meet the discharge readiness needs of this patient group.

## 1. Introduction

Oesophageal cancer represents a substantial global health burden, ranking as the 11^th^ most common malignancy and the seventh leading cause of cancer-related mortality worldwide (Bray et al., 2024). Data from the Global Burden of Disease 2021 study revealed a pronounced sex disparity, with the global incidence rate being significantly higher in males than in females (Liu et al., 2025). In 2022, approximately 511,000 new cases and 445,000 deaths were reported globally (Bray et al., 2024). Meanwhile, approximately 224,000 new cases and 187,500 deaths were reported in China, accounting for 44% and 42% of global cases, respectively (Han et al., 2024). Given that the overall five-year survival rate remains below 30%, this disease continues to severely strain the global healthcare system (Zhou et al., 2023).

Surgery, particularly radical resection combined with gastrointestinal reconstruction, remains the standard and most effective treatment for reducing patient mortality (Wang & Guo, 2023). However, esophagectomy is a highly invasive procedure associated with significant postoperative complications. Patients frequently experience a severe decline in quality of life, with postoperative complication rates ranging from 42.8% to 50.0% (Riba et al., 2019; Takeuchi et al., 2014). Consequently, postoperative mortality can exceed 5% within the first month and increase to 13% by three months post-surgery owing to complications (Mohiuddin & Low, 2015). In addition to increasing readmission rates, complications escalate economic burdens, strain medical resources, and critically impair the rehabilitation process.

Recently, the widespread adoption of Enhanced Recovery After Surgery (ERAS) protocols has transformed perioperative care. ERAS protocols have effectively reduced complication rates and significantly shortened postoperative hospital stays (Barrie et al., 2020; Grimminger, 2024). In 2019, guidelines were published to provide standardised recommendations for ERAS protocols in esophagectomy (Low et al., 2019). A systematic review of eight studies (seven from Western and one from China) indicated that ERAS contributed to reductions in pulmonary complications and hospital stay without increasing readmission rates (Markar, Karthikesalingam, & Low, 2015). However, the accelerated discharge facilitated by ERAS inherently shifts the burden of continuous recovery and complex symptom management from the hospital to the home environment (Barrie et al., 2020; Grimminger, 2024). The transitional challenges following esophagectomy are uniquely demanding compared with those associated with other surgical procedures. Patients must adapt to permanent alterations in gastrointestinal anatomy, which often require the management of feeding tubes and radically modifying their lifelong eating habits. A recent European study reported that even six to eight months post-discharge, patients suffer from debilitating symptoms, such as uncontrolled diarrhoea and severe nutritional difficulties, leading to social isolation and heavy reliance on family caregivers (Heramb-Aamot et al., 2024). These findings highlight the urgent need to ensure that patients are adequately prepared before hospital discharge.

The concept of "discharge readiness" provides a vital framework for addressing these transitional vulnerabilities. First conceptualised by Fenwick, (1979), discharge readiness involves both clinical assessment by healthcare professionals, including evaluations of physiological, psychological, and social factors, and the subjective perception of patients and their families regarding their capability to manage home care. One study indicated that higher levels of discharge readiness were strongly correlated with improved postoperative quality of life, reduced readmission rates, fewer complications, and a lower overall disease burden (Galvin et al., 2017). Despite its recognised importance, current studies on discharge readiness among patients with cancer predominantly rely on quantitative assessments. However, there is a distinct lack of qualitative research exploring the lived experiences and specific transitional needs of patients undergoing esophagectomy.

Therefore, this descriptive qualitative study aimed to explore the real-world experiences, multidimensional needs, and perceived discharge readiness of these patients. These findings provide a critical foundation for clinicians and nurses to develop personalised, evidence-based discharge interventions that optimise continuity of care.

## 2. Methods

### 2.1 Study design

A descriptive qualitative study was conducted. This study was conducted in accordance with the Consolidated Criteria for Reporting Qualitative Research (COREQ) (Tong et al., 2007).

### 2.2 Participants

The participants were recruited between July and September 2025 from Tianjin Medical University Hospital Cancer Institute & Hospital, a large academic hospital specialising in cancer treatment, care, and research located in Tianjin, China. Purposeful sampling was used to recruit patients from this tertiary-grade hospital in Tianjin, China. Age, educational level, sex, and type of surgical approach (open or minimally invasive) were considered during sample selection according to the principle of maximum difference variable (Penner et al., 2012). The sample size was determined based on data saturation, which occurs when continued data collection of new data (such as interviews and observations) no longer generates important new insights or information (Luciani et al., 2019; Patton, 1999).

The inclusion criteria for this study were as follows: 1) Adult patients (aged ≥ 18 years) diagnosed with oesophageal malignancy who underwent radical esophagectomy; 2) Demonstrated normal cognitive function and communication abilities; 3) About to be discharged from the hospital; 4) Aware of their cancer diagnosis. The exclusion criteria were as follows: 1) A history of psychiatric disorders, 2) Presence of concurrent malignancies in other organs or severe organic diseases, and 3) Diagnosis of conditions impairing cognitive function, such as Alzheimer’s disease.

### 2.3 Data collection

In this study, all interviews were conducted by a female researcher (YXW), a nurse with a master’s degree qualification and one year of work experience in the oesophageal cancer department. She had received extensive theoretical expertise in qualitative research and professional interview training during her master’s programme. None of the participants were known to the interviewer prior to the study.

Semi-structured face-to-face interviews were conducted in accordance with the interview guidelines. Each session was recorded using a digital voice recorder. Interviews were scheduled within 24h prior to discharge and were conducted in a private conference room to ensure a comfortable, quiet, and undisturbed environment. During the interviews, the researcher posed questions based on the guide while encouraging participants to fully express their feelings. Timely responses were provided without leading, hinting, or arbitrarily interrupting participants' thought processes. Simultaneously, non-verbal behaviours were observed, and changes in facial expressions, tone, pitch, emotions, and body language were objectively documented. Each interview lasted from 30 to 45 minutes. The phrasing and sequence of the questions were flexibly adjusted based on the actual flow of the interview to maximise information collection and ensure that the conversation remained focused on the research objectives.

#### 2.3.1 Interview guide

An interview guide was formulated based on the study objectives. Pilot interviews were conducted with two patients who had undergone radical resection and were preparing for discharge. Based on the practical outcomes of these pilot interviews and the feedback received from the patients, the research team conducted discussions to refine the interview content. A detailed version of the interview guide is provided in Appendix A.

#### 2.3.2 Research team

The research team comprised experienced nursing experts, including three core researchers. The three researchers were responsible for the study design, interview execution, and data analysis. All interviews were conducted collaboratively by two researchers: one facilitated the interview and the other conducted field observations. Furthermore, all team members had received formal training in qualitative research methods and possessed extensive clinical nursing experience, thereby ensuring the methodological rigour and scientific validity of the study.

### 2.4 Data analysis

Data were analysed using Colaizzi’s seven-step phenomenological analysis method (Hsieh & Shannon, 2005): 1) Transcribing all participants' descriptions: all interview protocols were read repeatedly to acquire a feeling for them. 2) Extracting significant statements: Two researchers independently analysed the transcripts and extracted phrases directly pertaining to the investigated phenomenon. 3) Formulating meanings: the two researchers independently summarised and refined the meanings of these significant statements to formulate ideas that identified common characteristics. 4) Clustering themes: The researchers combined their findings and aggregated the formulated meanings into thematic clusters. 5) Developing an exhaustive description: all themes were linked to the original interview data to generate a comprehensive description of the phenomenon. 6) Identifying final themes: the essential structure of the phenomenon was distilled to determine the final themes. 7) Seeking verification from participants: the analysis results were returned to the participants for validation and potential modifications to ensure accuracy. All interview data were transcribed verbatim and analysed using Nvivo 11.0 software. Throughout the data analysis process, two researchers (YXW, YJX) were responsible for reading, organising, and analysing the data independently. Any discrepancies or ambiguous results were resolved through discussion with a third researcher (YW) until consensus was achieved. This procedure ensured the credibility and reliability of the results.

### 2.5 Ethical considerations

This study was reviewed and approved by the Ethics Committee of Tianjin Medical University Cancer Hospital (bc20255765). Prior to the formal interviews, the researchers introduced themselves and elucidated the purpose, content, and significance of the study to the patients. The necessity of audio recording was also explained. Participants were assured of strict adherence to the principles of confidentiality and anonymity. After reading the information letter, 12 patients provided written informed consent to participate in the study.

### 2.6 Scientific rigour

To ensure trustworthiness, all interviews were conducted by the principal researcher, while another researcher consistently observed and documented non-verbal information in a field diary. We ensured that the researchers had no prior relationship with the participants. All study procedures, including the interview guide, standardised data collection, data transcription, and data analysis, were carefully documented to ensure the dependability of the study. Multiple techniques, including data saturation, researcher triangulation, and member checking, were used to ensure methodological rigour and validity. Appropriate interview techniques and reflexive practices were applied to ensure the confirmability of the study. The use of neutral interview techniques maximised the patients’ opportunities to express their ideas honestly and comprehensively. The COREQ checklist guided the reporting of the methodological and analytical procedures used in this study.

## 3 Results

### 3.1 Participants’ characteristics

A total of 12 patients with oesophageal cancer were included in this study (2 females and 10 males), with no dropouts. Their ages ranged from 45 to 68 years. Nine patients underwent robotic-assisted esophagectomy. Details of the participants are presented in Table I. No postoperative complications or ICU admissions occurred during hospitalisation. While there was a noticeable sex imbalance within the sample, this reflects the established epidemiological reality of oesophageal cancer in China. To protect the privacy of the respondents, their names were omitted and replaced with the numbers P1 to P12. Repeat interviews were not conducted.

**Table I.** Demographic information of patients.

Number ID	Age	Gender	Marital status	Educational degree	Type of occupation	Approach of surgery	Comorbidities	Family monthly income (yuan)
**1**	62	male	married	middle school degree	retired	open esophagectomy	None	≤5000
**2**	60	male	married	middle school degree	retired	thoracolaparoscopic esophagectomy	None	≤5000
**3**	65	male	widowed	under middle school degree	farmer	robot assisted esophagectomy	None	≤5000
**4**	58	male	married	associate degree	self-employment	robot assisted esophagectomy	None	5001–7000
**5**	65	male	divorced	associate degree	retired	robot assisted esophagectomy	hypertension	≤5000
**6**	58	female	married	middle school degree	retired	robot assisted esophagectomy	None	5001−7000
**7**	48	male	married	bachelor degree	civil servant	robot assisted esophagectomy	diabetes	5001−7000
**8**	62	male	married	under middle school degree	farmer	robot assisted esophagectomy	None	≤5000
**9**	56	female	married	middle school degree	unemployed	robot assisted esophagectomy	None	≤5000
**10**	68	male	married	bachelor degree	retired	robot assisted esophagectomy	hypertension	≤5000
**11**	64	male	married	bachelor degree	retired	robot assisted esophagectomy	None	≤5000
**12**	45	male	married	middle school degree	civil servant	robot assisted esophagectomy	None	7001–8000

### 3.2 Qualitative results

Three themes and eight subthemes were generated from the data.

#### 3.2.1 Theme 1: coexistence of positive and negative emotions regarding discharge readiness

The discharge preparation period was a dynamic process characterised by emotional ambivalence. Patients frequently experienced both positive and negative emotions simultaneously. Positive feelings primarily stemmed from relief after esophagectomy, visible rehabilitation progress, and a strong desire to return to a familiar home environment. Conversely, negative emotions arose from fears of disease recurrence and a sudden sense of vulnerability associated with the loss of continuous professional medical support. This coexistence of emotions represented a typical psychological response during the health transition process rather than a sign of individual maladaptation.

##### 3.2.1.1 Subtheme 1: expression of positive emotions at discharge.

Following the successful completion of surgery and postoperative treatment, along with confirmation via radiological assessment that the discharge criteria had been met, a subset of patients expressed joy regarding their rehabilitation and return home.

*“I felt in good condition now. After the initial chemotherapy and the completion of the surgery, the next step was just to go home and recuperate. I was confident in my full recovery.”*(P1) *“The nursing care was excellent, and you provided a lot of useful guidance, which was very helpful for our rehabilitation at home.”*(P3) *“I was originally a bit worried and nervous before the surgery, but I improved day by day postoperatively. I was very happy to be discharged home so soon.”*(P7)

##### 3.2.1.2. Subtheme 2: expression of negative emotions at discharge.

Esophagectomy is a highly complex procedure requiring significant gastrointestinal reconstruction. As a result, patients experienced a prolonged recovery period. The reality of leaving the hospital triggered immediate anxiety, fear, and uncertainty regarding their ongoing physical condition and the potential risk of cancer recurrence.

*“I wanted to know when the pathology results would be available and if I needed to continue chemotherapy. I did not know when I could return to normal life.”*(P5) *“A fellow patient suffered metastasis less than three months after discharge. Would I experience the same situation?”*(P9) *“Would my voice ever be restored to its preoperative state? I have suffered from voice hoarseness the completion of surgery.”*(P10)

#### 3.2.2 Theme 2: perceived dilemmas regarding discharge readiness

In addition to immediate emotional reactions, patients also faced profound internal and cognitive dilemmas. The persistence of symptoms such as abdominal distension, diarrhoea, and pain complicated the recovery process. These challenges led to internal conflicts regarding discharge decisions, social identities, and health literacy.

##### 3.2.2.1 Dilemmas in discharge decision-making.

Patients struggled with the actual decision to leave the hospital. Most patients felt unprepared for the transition home because they required continuous enteral nutrition and experienced distressing postoperative symptoms. Harbouring doubts about their recovery status, these patients expressed a desire to extend their hospitalisation to avoid potential complications, such as anastomotic fistulas.

*“I did not want to be discharged yet. I had a fever the day before yesterday and would prefer to stay for a few more days for observation. I was unsure how to manage it if the fever recurred after I go home.”*(P7) *“The doctor told us to discharge, but I was unaware of the extent of my recovery or whether I have truly met the discharge criteria.”*(P11) *“I have not yet adapted to the nutrient solution and constantly felt abdominal distension. Could I stay a few more days to adjust? I was from out of town, so it would be very inconvenient to return to the hospital if problems arose at home.”*(P6) *“I would like to rest in the hospital for a few more days; I was worried that going home too early might lead to an anastomotic fistula.”*(P12)

##### 3.2.2.2 Dilemmas of role conflict.

Some patients were in the prime of their lives and bore heavy family responsibilities, making them eager to resume their roles as economic providers or caregivers. However, post-esophagectomy physical limitations, such as the need to manage feeding tubes, severe gastroesophageal reflux, and voice hoarseness, created massive barriers to social interaction and employment. This stark contrast between their desired social roles and their physical condition resulted in significant maladaptation.

*“My voice was still very hoarse. How long would it take to recover after discharge? It significantly affected my communication with others, making it impossible to resume work.”*(P10) *“If I went out now, everyone can tell I have been sick and had surgery; the scar was very obvious. [sighs] I did not want to be looked at differently.”*(P4) *“The treatment costed a significant amount of money. Even after surgery, I could not earn money to support my family and feel like a burden to the whole household. [Lowered head]”*(P12)

##### 3.2.2.3 Dilemmas regarding disease-related knowledge needs.

Patients faced a critical gap between the knowledge required for recovery and their actual understanding of post-discharge care. Due to limited educational attainment or insufficient communication, some patients exhibited severe deficits in disease-related knowledge. Consequently, they relied entirely on family members to manage post-discharge care protocols and follow-up schedules, thereby increasing uncertainty regarding their prognosis.

*“I do not know what needs to be done after returning home. The doctor gave me a rough idea, but I am unclear about the specifics.”*(P5) *“I just listen to her [my wife]; I did not memorize the specific details.”*(P8) *“I am unclear about the follow-up appointment time. The doctor did not tell me directly; they spoke to my children instead.”*(P2)

#### 3.2.3 Theme 3: multidimensional challenges in post-discharge home care

Post-esophagectomy patients faced multifaceted challenges during the hospital-to-home transition. First, patients often lacked adequate self-care competence, which diminished their confidence and increased their reliance on caregivers. Second, family support was frequently constrained; primary caregivers experienced role conflicts (e.g., balancing work and parenting) that limited their time and energy to provide continuous care. Finally, inadequate community and online transitional support left patients struggling to access essential home-based medical care, wound management, and symptom management services. Collectively, these personal and systemic barriers significantly hindered a smooth transition to home-based recovery.

##### 3.2.3.1. Insufficiency of self-care competence.

Post-esophagectomy, patients required advanced skills to manage their ongoing care, particularly home-based enteral nutrition. However, patients demonstrated suboptimal mastery of these technical skills. This lack of practical competence diminished their confidence in self-management and resulted in substantial dependence on their primary caregivers.

*“Tube blockages during hospitalization were resolved by nurses. I was unsure if I can handle it myself if it occurred at home.”*(P2) *“As you mentioned, I had little education and did not understand these things well; I would rely entirely on my children after returning home.”*(P8) *“I knew that I could not lie flat while sleeping to prevent reflux, and I needed to continue administering nutrient solution into my stomach and flush the tube regularly to prevent blockages. However, my spouse has learned how to flush the tube; I did not know how to do it myself, so I would rely entirely on her at home.”*(P10)

##### 3.2.3.2. Constraints of the family support system.

Because patients lacked self-care competence, they relied heavily on family support, which was often structurally constrained. Primary caregivers frequently occupied multiple societal roles and balanced responsibilities as employees, parents, and spouses. This role conflict meant that caregivers lacked the continuous time, energy, and specialised medical capacity required to meet the patients' complex needs.

*“My children had their own families to take care of, and my spouse had passed away. Since I was getting old, these procedures [enteral nutrition pumping, oral feeding requirements, etc.] were too complex for me. I could not take care of myself and could only hire a care worker.”*(P3) *“My son needed to work to support the family and pay for my medical treatment; the chemotherapy and surgery had costed a lot. He could not take leave frequently to care for me. Additionally, my grandson needed to be looked after. I was divorced and have been living alone; my son could only come to help on weekends.”*(P5)

##### 3.2.3.3. Inadequacy of the transitional care support system.

Patients universally expressed a desire for timely support and assistance from medical personnel after returning home, indicating a high demand for home-based medical care and community nursing services. The current lack of community-based professional resources further exacerbated these vulnerabilities.

*“After discharge, it would be ideal if nursing staff could visit daily to administer the nutrient solution. We were not professionals; what should we do if we encounter problems we could not handle?”*(P1) *“I hoped a WeChat group can be established so that we could contact doctors and nurses in a timely manner if questions arose.”*(P3) *“I was unsure if the local hospital could handle wound dressing changes and suture removal. What should I do if diarrhea or fever recurs? It was too far to come back here; I hoped to receive some professional guidance online.”*(P7) *“I hoped you can provide a specific plan regarding daily diet and activity, and helped us transition for a week after returning home; that way, I would master it.”*(P9)

## 4. Discussion

The current study illuminated the profound complexities faced by patients with oesophageal cancer during the hospital-to-home transition. Rather than representing a simple change in physical location, discharge from the hospital constituted a highly vulnerable period characterised by psychological, social, and functional disruption. Our findings emphasised that the evaluation of discharge readiness must extend beyond basic clinical checklists to encompass a holistic assessment of patients’ psychosocial adaptability and the structural capacity of their home support systems.

Our findings revealed that the psychological experience of discharge was not a linear progression toward relief, but rather a volatile coexistence of positive and negative emotions. This emotional ambivalence requires critical interpretation. The anticipation of returning home often boosts patients' self-efficacy, aligning with the concept of an internal health locus of control (Rock et al., 1987), in which perceived physical improvement empowered proactive rehabilitation behaviours. However, this optimism was heavily counterbalanced by the chronic dread of disease recurrence and the sudden withdrawal of the hospital's "safety net." Consistent with the findings of Hellstadius et al. (2017) and Housman et al. (2021), this persistent distress was heavily associated with the fear of independent management of potentially life-threatening complications. This emotional change underscored that psychological distress at discharge was not a sign of individual maladaptation, but rather a systemic transition shock. Therefore, nurses must shift from providing purely instructional discharge education to delivering transitional psychological support. Clinicians should explicitly validate and normalise this emotional ambivalence to ensure that patients do not perceive their fear as a personal failure. Implementing structured psychological debriefing sessions prior to discharge could mitigate this anxiety.

A critical and somewhat unexpected finding in our study was the severe deficit in disease-related knowledge among patients, which contributed to profound hesitancy regarding discharge. This phenomenon cannot be attributed solely to low educational attainment; rather, it should also be interpreted within a cultural perspective. It is deeply rooted in traditional Chinese "protective medical practices" (Yan et al., 2025). Within this cultural norm, families often shield patients from unfavourable prognostic information or complex medical details, inadvertently reducing patients’ autonomy in the decision-making process. Consequently, patients demonstrated limited self-management capability and delayed adaptation to the "patient role" because they are structurally excluded from their own care planning. Furthermore, the physical limitations imposed by esophagectomy, such as voice hoarseness and reliance on feeding tubes, precipitated severe role conflicts, particularly among middle-aged male patients traditionally identified as primary economic providers. The inability to fulfil these societal and familial expectations further exacerbated their psychological burden, consistent with the findings reported by Markar et al. (2015). Healthcare professionals should recognise that patient education within this cultural context is inherently a family-centred issue. Clinical practice should implement culturally sensitive, triadic (nurse-patient-family) shared decision-making models. Nurses should tactfully navigate familial protective instincts and facilitate open dialogue that empowers patients with essential disease-related knowledge while respecting family dynamics. In addition, occupational therapy interventions should be integrated early in the postoperative phase to help patients navigate their altered social identities.

The multidimensional challenges observed in post-discharge home care revealed severe fragmentation between surgical care and community support services. The sheer complexity of post-gastrointestinal reconstruction care (e.g., enteral nutrition management and severe reflux prevention) heavily burdened the family support system. As primary caregivers juggle multiple societal roles (employees and parents), their capacity to provide continuous specialised care was often overwhelmed, consistent with the caregiver burden paradigms highlighted by Muliira et al. (2019) and Yoshida et al. (2020). The pervasive demand for professional home-based support in our study, paired with evidence that post-esophagectomy struggles can persist for months (Heramb-Aamot et al., 2024), indicates that traditional one-time discharge instructions are fundamentally inadequate. Healthcare institutions should therefore develop robust technology-driven transitional care networks to address this critical gap. Yu et al. (2022) demonstrated that proactive interventions can significantly improve postoperative outcomes. Accordingly, we strongly advocate the development of localised app-based digital health platforms that provide interactive symptom tracking, video-based enteral tube management tutorials, and asynchronous telehealth consultations. Furthermore, policy-level interventions are necessary to strengthen tiered healthcare systems. Acute care hospitals must establish formal collaborative referral pathways with community health service centres and provide specialised training for community nurses to safely manage wound care and tube complications within local communities.

This study has several limitations. First, although maximum variation sampling was used, all participants were recruited from a single tertiary hospital in northern China; thus, regional healthcare disparities and local cultural nuances may have limited the transferability of the findings. Second, a vast majority of our patients underwent robot-assisted esophagectomy. Given the differences in surgical trauma and postoperative recovery trajectories (Wu et al., 2024), the discharge experiences of patients undergoing open procedures may not have been fully captured. Third, this cross-sectional qualitative study interviewed patients immediately before discharge to capture their anticipated needs and perceptions regarding discharge readiness. Future studies should employ longitudinal qualitative designs to track how discharge readiness translates into long-term coping mechanisms. Furthermore, the sample exhibited a significant gender imbalance. Although this disproportion naturally aligns with the higher prevalence of oesophageal cancer among men, it limited the thorough exploration of sex-specific experiences during the hospital-to-home transition. Previous research has indicated that men and women with gastroesophageal cancer often experience distinct sociocultural pressures, caregiver dynamics, and psychological burdens (Dijksterhuis et al., 2021). For instance, the severe role conflicts and caregiver dynamics observed in this study were heavily influenced by the male perspective, particularly regarding the loss of the primary economic provider’s identity. Female patients may face entirely different societal expectations, family support structures, and psychological burdens, which this study could not fully capture. Future studies should adopt multicenter, cross-cultural designs with larger and more diverse samples, including more female participants and different surgical options, to ensure a more comprehensive understanding of discharge readiness to enhance the clinical applicability and generalisability of the results.

## 5. Conclusions

During the hospital-to-home transition, post-esophagectomy patients experienced complex emotional distress, significant self-care deficits, and profound role conflicts. To effectively enhance discharge readiness, personalised intervention strategies for different patients should include structured training in enteral nutrition management, targeted psychosocial counselling, the establishment of digital transitional care, and the strengthening of community healthcare support. This study emphasises the importance of continuous nursing care in promoting discharge readiness and provides a useful reference for future clinical practice.

## Data Availability

Data is provided within the manuscript or supplementary information flies.

## Appendix A

**Domain 1: Overall experience and perception of self-care requirements**

1. Please tell me about your recent experience regarding the esophagectomy and your postoperative recovery.

- *e.g., How are you feeling physically today? What has been the most uncomfortable or challenging part of your recovery (e.g., feeding tube management, gastroesophageal reflux, voice hoarseness)?*

2. What conditions do you think are necessary to possess self-care ability after discharge?

- *e.g., In terms of physical strength, symptom control (like pain or reflux), specific nursing skills (like flushing the feeding tube), or psychological readiness, what must be in place for you to manage safely at home?*

**Domain 2: Perception of discharge readiness and emotional responses**

3. What were your initial thoughts and inner feelings when you were informed of your impending discharge?

4. Based on your current condition, do you feel you are fully ready to be discharged from the hospital?

- *If yes:* What makes you feel confident? What do you think went well in your preparation?
- *If not/unsure:* What are your main concerns or worries?
- *e.g., Fear of complications like anastomotic fistula or fever, uncertainty about recovery, anxiety about leaving the hospital's safety net.*

**Domain 3: Self-care competence and disease-related knowledge**

5. What disease-related knowledge and post-operative skills have you acquired so far from the care team?

- *e.g., Do you feel confident in managing home enteral nutrition, flushing the feeding tube, preventing reflux during sleep, and knowing your follow-up schedule?*

6. What specific information, skills, or instructions do you feel you still need to understand better before leaving the hospital?

7. Do you feel like the hospital staff took your and your family's actual comprehension levels and preferences into account when explaining the discharge plan?

**Domain 4: Family support and transitional care needs**

8. Can you get sufficient help from family members once you are home?

- *e.g., Do they have the time, energy, and skills to support you? Are they able to handle financial burdens, provide emotional support, or assist with complex personal care like feeding tubes?*

9. What professional support or assistance do you hope to receive from healthcare providers after discharge?

- *e.g., Convenient acquisition of home-based medical services, wound dressing changes in the community, online symptom management guidance (like a WeChat group), or telephone follow-ups.*

**Domain 5: Feedback and recommendations**

10. If you were making recommendations to the hospital to improve the discharge preparation process for future esophagectomy patients, what would you suggest?

- *e.g., What was helpful and should stay the same? What should be done differently? Were there any needs that were ignored?*
